# Advancing acute MI care in densely populated low- and middle-income countries (LMICs): innovative stand-alone chest pain units for expedited triage and timely management

**DOI:** 10.1016/j.lansea.2024.100488

**Published:** 2024-09-30

**Authors:** Nadeem Qamar, Jawaid A. Sial, Tahir Sagir, Zair Hussain, Ali Zain Shah, Kamran Khan, Jehangir Ali Shah, Musa Karim, Shueeta Kumari, Sohail Khan, Sabha Bhatti, Abdul Hakeem

**Affiliations:** National Institute of Cardiovascular Diseases, Rafiqui Shaheed Road, Karachi, Pakistan

**Keywords:** Myocardial infarction, Triage, Percutaneous coronary intervention

## Abstract

**Background:**

The incidence of myocardial infarction (MI) and its adverse effects on health and mortality remain high in densely populated low- and middle-income countries (LMICs). To address the issue of densely populated areas and timely access to primary PCI, chest pain units (CPUs) were deployed at strategic locations in Karachi, with a populace of over 23 million people. This study describes the results of this initiative in expediting MI care.

**Methods:**

Between 2017 and 2023, 18 CPUs, each with a cardiologist, technician, ECG machine, crash cart and an ambulance were placed in high density areas.

**Findings:**

A total of 915,564 patients were seen at 18 CPUs over the study period. 692,444 (75.6%) were categorized as non-cardiac and subsequently discharged. 223,120 (24.6%) patients were directed for additional care. Of these, 9% had ST elevation myocardial infarction (STEMI) (19, 580), 29% NSTE ACS/Unstable angina, and 31% with various other cardiac conditions. Additionally, 31% were referred for medical outpatient evaluation. CPU inception led to a significant annual growth (16–20%) in primary PCI procedures at NICVD, totaling 20,000 by 2022–2023. The median first medical contact to device time was 100 min (IQR 80–135), while total ischemic was 232 min (IQR: 172–315; 5th −95th %le: 50–920). The overall in-hospital mortality rate for patients undergoing primary PCI was 5.58%, with a range between 5.1% and 6.9% through the study period.

**Interpretation:**

Novel standalone chest pain units, operational from 2017 in Karachi, Pakistan, have expedited triage and enhanced the timely management of AMI. This initiative's transformative impact presents a model that resonates beyond borders, serving as a role model for global healthcare systems.

**Funding:**

The CPU and primary PCI program is fully funded by the government of Sindh. No specific funding was allocated for this study.

**Research in context**

**Evidence before this study**

While rapid access chest pain centers in the Western world play a crucial role in expedited acute myocardial infarction care by streamlining emergency room flow, they primarily operate within hospital premises. This practice of admitting a large number of chest pain patients often leads to overtriage and increased healthcare costs. Therefore, developing innovative chest pain units that focus on efficient triage and prompt treatment of acute cardiac complaints is essential to enhance patient outcomes and optimize resource utilization.

**Added value of this study**

NICVD's innovative implementation of stand-alone Chest Pain Units represents a paradigmatic shift in acute MI care within a densely populated setting in LMICs. These CPUs strategically placed in critical locations within a densely populated metropolis such as Karachi facilitated timely access for urgent evaluation, catering to a substantial cohort of 0.92 million patients over a 6-year time period. This approach proved highly effective in screening and triaging patients, resulting in an unprecedented surge in ST cases and primary PCI volumes within the NICVD system. Furthermore, the identification of approximately 5% of patients with NSTE ACS, including unstable angina, underscores the CPUs' efficacy in recognizing diverse cardiac pathologies beyond the typical STEMI presentations.

**Implications of all the available evidence**

The CPU model's impact extends beyond traditional rapid access centers by offering immediate ECG access and streamlined referrals, addressing the unique challenges of densely populated LMICs. This initiative serves as a potential blueprint for global AMI care, ensuring equitable access and improved outcomes. This approach, rooted in strategic placement and timely management, meets the healthcare challenges of South and East Asian nations and encourages global healthcare systems to rethink their strategies.

## Introduction

Cardiovascular diseases (CVD), including acute myocardial infarction (AMI), constitute a substantial global health challenge, particularly in low- and middle-income countries (LMICs) such as Pakistan. The South Asian region, including Pakistan, carries the highest burden of coronary artery disease, with alarming rates of AMI occurring even among individuals in their 40s and younger.^1^ According to the 2019 Global burden of disease study, incidence of CVD in Pakistan was 918/100,000 (global 684/100,000) and age standardized death rate was 358/100,000 (global 240/100,000). Furthermore, the epidemiology of CVD within South Asia is heterogeneous such that Pakistan had the highest ischemic heart disease mortality and disability adjusted life-years in the entire South Asian region.^1^ Amongst various factors outlined, timely access to primary percutaneous coronary intervention (PCI) remains one of the most important factors driving this high mortality.^2^^,^^3^ Importantly, AMI tends to affect the young (mean age of patients, 52.2 ± 10.7 years with 28.3% patients <45 years) leaving a huge toll on the family and society in terms of disability and quality adjusted life years (DALYS and QALYS).^4^^,^^5^^,^^6^

Karachi, with a population exceeding 23 million, grapples with the complexities of providing timely cardiac care.^2^ Recognizing the critical need to enhance access to primary PCI services, a pioneering effort was undertaken to establish 18 stand-alone chest pain units (CPU) across the city. This initiative aimed to expedite the evaluation and referral of AMI patients to the National Institute of Cardiovascular Diseases (NICVD), that has consequently become the world's largest primary PCI center.^2^

This article outlines the establishment and functioning of NICVD's integrated CPU model, highlighting its key components, including patient demographics, clinical profiles, time intervals, and primary reperfusion features. It examines the challenges encountered during implementation and operation, and suggests potential enhancements for the future. Furthermore, this study evaluates the impact of the CPU model on effective screening and triage of patients with cardiac complaints, as well as the number of primary PCIs performed over the years. The primary aims are to describe the introduction of the CPU model and evaluate its initial period of operation, emphasizing how these units improve patient access, outcomes, and reduce total ischemic time.

## Methods

This descriptive study delineates the initiation, implementation and expansion of the novel chest pain units’ program by NICVD.

One of the pivotal determinants influencing the door-to-balloon (DTB) time and subsequent timely reperfusion for the ST elevation myocardial infarction (STEMI) patient is the financial dimension of healthcare. Access to primary PCI is often impeded for patients who lack the necessary financial resources and health insurance, rendering them exceedingly vulnerable in urgent circumstances. Prior to 2016, individuals in Karachi experiencing STEMI could undergo primary PCI if covered by health insurance or possess the financial means to pay for the procedure themselves (<2% of the population). In 2016, the NICVD, the pre-eminent cardiac hospital in the region under the auspices of the Government of Sindh, instituted a program offering free primary PCI. In the inaugural year of this initiative, the number of primary PCIs more than doubled compared to prior years from 1500 to 2000/year to more than 4000 cases in 2016.

However, the program's nascent phase brought to light several challenges, particularly in the dynamic and densely populated metropolis setting of Karachi whose population exceeds 23 million, translating to a population density surpassing 24,000 individuals per square kilometer or 63,000 per square mile. This renders Karachi (along with Dhaka and Mumbai) significantly denser than any other designated “megacity” with an urban populace exceeding 10 million. In this environment, challenges encompass intricate road access, substantial congestion in emergency departments (ED) handling a daily caseload exceeding 1000 patients at NICVD alone, and formidable impediments in ensuring expeditious diagnosis and access for the residents of this densely populated metropolis.

To address these multifaceted challenges, enhanced public awareness and swift assessment and triage of high-risk patients presenting with acute chest pain were utilized to implement the innovative paradigm of stand-alone CPUs. Multiple units were strategically established in key areas characterized by the highest population density across the city.

### Infrastructure and operations

A typical CPU is comprised of a container housing essential components, including a bed, a computer, a crash cart equipped with a defibrillator and crucial medications such as antiplatelet therapy and heparin. Each CPU is staffed by a qualified cardiologist with a minimum of a two-year diploma in cardiology, emphasizing adult cardiology and emergency cardiac care. The dedicated team also includes a cardiac nurse, technical staff, and an ambulance driver provided by Edhi services, recognized as the world's largest ambulance network. These units operate around the clock, 365 days a year (Supplemental Fig. S1a and b; Video).

**Fig. 1:**
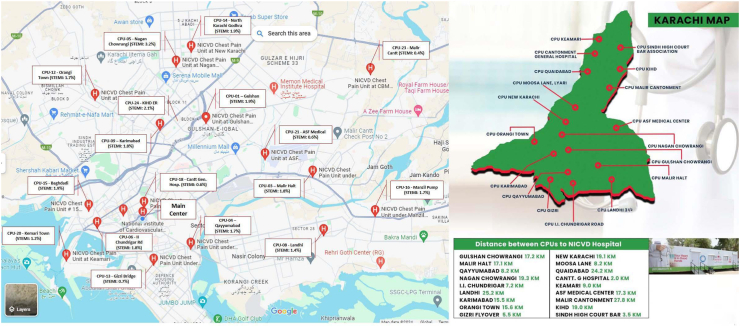
Maps of Karachi showing distribution of the chest pain units relative to the main center. Driving distance from CPUs to the main center.

Fig. 1 shows the map of Karachi and the location of CPUs across the city with travel distance to the main campus. The closest CPU is 2 kms from the NICVD, while the farthest is 27.8 kms, with a median distance of approximately 15 kms. The average commute time is around 35 min. Most patients are transported via ambulance to ensure timely transfer for PPCI. Some patients also arrive at CPUs by walk-in, taxi, or other means, but emergency transfers to NICVD are predominantly managed by ambulance services to facilitate swift and safe transit.

### Operational algorithm

The operational algorithm of the CPU involves patient screening through a comprehensive history and physical examination, followed by a prompt 12-lead ECG in keeping with current guidelines.^7^ Patients are categorized into three groups based on a comprehensive assessment, including history, ECG, physical examination, and vital signs. STEMI patients receive immediate treatment, including dual antiplatelet therapy (DAPT) and heparin, before being swiftly transferred to the main campus via ambulance. Upon reaching the main campus, those in Killip I/II are directly conveyed to the cath lab based on availability, while those in Killip III/IV are stabilized in the ED before proceeding to the cath lab for Primary PCI.

Patients with normal ECG and clear non-cardiac chest pain are discharged after receiving counseling on preventive measures. Those with typical cardiac chest pain symptoms and a history consistent with unstable angina or ischemic ECG changes are urgently transferred to the main hospital. Patients with stable angina or suspected cardiac disease are referred to the outpatient clinics at the main NICVD center.

### Cath lab capacity

The main center houses a total of eight cath labs, with five dedicated to STEMI patients at any given time and all eight available at all hours for handling cardiac emergencies. A team of 30 interventional cardiologists serves as faculty, supported by 52 interventional cardiology fellows and 285 general cardiology fellows.

### Sources of data

Data from the Electronic Medical Records (EMR) of CPUs were collected and analyzed. Recorded variables encompassed age, sex, triage category, and disposition. Furthermore, data from the Cath Lab Management System spanning the study period from 2015 to 2023 were obtained. Essential variables, including estimates of total ischemic time, DTB and inpatient mortality, were retrieved for analysis.

### Data analysis

Data are summarized using appropriate descriptive statistics. Continuous variables are presented as mean ± standard deviation (SD) or median [interquartile range (IQR)], while categorical variables expressed as frequency (percentage).

The analysis of trends in the annual/quarterly volume of procedures over the years/quarters was performed by conducting joinpoint regression analysis using the Joinpoint Regression Program 7 (Version 5.0.2), developed by the “Statistical Research and Applications Branch of the National Cancer Institute”.^8^ We assumed homoscedasticity and uncorrelated error terms and fitted log-transformed model with data dependent model selection method. We report the average annual percentage change (AAPC) and its corresponding 95% confidence interval (CI) for the entire study period (2015–2023) and each segment within the final selected model. Univariable and multivariable binary logistic regression analyses were performed to identify factors associated with in-hospital mortality. Variables with plausible clinical associations, based on the available literature, were included in the univariable and multivariable analyses. The backward conditional variable selection method was used to finalize the multivariable model, employing IBM SPSS version 21. Statistical significance was determined at the 5% level.

### Expansion of CPU and primary PCI services across the entire province

Since its inception in 2016, the initiative has demonstrated notable success by establishing nine additional primary PCI centers and a total of 24 CPUs throughout the province of Sindh, with Karachi as its capital city (Supplemental Table S1). Covering an area comparable to England and serving a population exceeding 56 million, this strategic expansion is designed to enhance accessibility to cardiac care.

### Role of funding

There was no funding for this manuscript. The authors take full responsibility for the content and integrity of the presented data.

## Results

### Trends in patient volumes at CPUs

From the program's initiation in 2017 to 2023, a cumulative total of 915,564 patients received care at 18 CPUs. The inception of the first two CPUs, coupled with awareness campaigns and the introduction of the free Primary PCI program, triggered a remarkable upswing in case numbers (Table 1). To address the escalating demand, the number of CPUs expanded from an initial two to the current count of 18 CPUs distributed across the city.

**Table 1:** Characteristics of patients presenting to the CPUs∗

	Total patients	Gender		Age			Diagnosis		Disposition		STEMI∗
		Male	Female	≤40 years	41–65 years	>65 years	Cardiac	Non-cardiac	Referred	Discharge	
**Total**	915,564	564,129 (61.6%)	351,435 (38.4%)	352,872 (38.5%)	485,102 (53%)	77,590 (8.5%)	306,794 (33.5%)	608,770 (66.5%)	223,120 (24.4%)	692,444 (75.6%)	19,580 (2.1%)
2017	21,857	16,074 (73.5%)	5783 (26.5%)	8159 (37.3%)	11,823 (54.1%)	1875 (8.6%)	9815 (44.9%)	12,042 (55.1%)	9817 (44.9%)	12,040 (55.1%)	1329 (6.1%)
2018	114,093	71,411 (62.6%)	42,682 (37.4%)	45,234 (39.6%)	59,740 (52.4%)	9119 (8%)	40,295 (35.3%)	73,798 (64.7%)	29,133 (25.5%)	84,960 (74.5%)	3626 (3.2%)
2019	187,814	114,217 (60.8%)	73,597 (39.2%)	72,565 (38.6%)	98,970 (52.7%)	16,279 (8.7%)	55,257 (29.4%)	132,557 (70.6%)	37,296 (19.9%)	150,518 (80.1%)	2527 (1.3%)
2020	135,743	85,949 (63.3%)	49,794 (36.7%)	50,965 (37.5%)	73,031 (53.8%)	11,747 (8.7%)	49,482 (36.5%)	86,261 (63.5%)	34,700 (25.6%)	101,043 (74.4%)	2049 (1.5%)
2021	164,154	102,949 (62.7%)	61,205 (37.3%)	65,000 (39.6%)	85,393 (52%)	13,761 (8.4%)	58,753 (35.8%)	105,401 (64.2%)	41,847 (25.5%)	122,307 (74.5%)	2688 (1.6%)
2022	202,957	119,959 (59.1%)	82,998 (40.9%)	76,639 (37.8%)	109,164 (53.8%)	17,154 (8.5%)	70,376 (34.7%)	132,581 (65.3%)	50,244 (24.8%)	152,713 (75.2%)	5022 (2.5%)
2023	88,946	53,570 (60.2%)	35,376 (39.8%)	34,310 (38.6%)	46,981 (52.8%)	7655 (8.6%)	22,816 (25.7%)	66,130 (74.3%)	20,083 (22.6%)	68,863 (77.4%)	2339 (2.6%)
**Average Annual Percentage Change (AAPC) [95% confidence interval]**											
Full Range	27.5 [1.3–55.6]	24 [1.5–47.7]	34.8 [8.4–62.2]	27.7 [−1.8 to 60.7]	27.3 [2.9–53]	27.9 [3.7–53.7]	20.2 [−14.8 to 62.6]	31.8 [6.5–58.8]	17.2 [−13.8 to 53.3]	33.1 [8.5–58.6]	10.1 [−11 to 34.3]
2017–2019	180.2 [46.3–463.1]	159.4 [43.3–377.5]	230.3 [62.7–527.8]	183.2 [43–539.2]	177.7 [48.6–427.4]	182.3 [51.4–438.9]	146.6 [−8.3 to 609.1]	204.5 [58.2–488.2]	101.5 [−13.3 to 414.2]	228.6 [64.9–504.2]	27.4 [−30.1 to 148]
2019–2023	−14 [−53.4 to 7.5]	−14.3 [−49.3 to 4.7]	−13.8 [−50.4 to 10.3]	−14.3 [−59.7 to 10]	−13.8 [−50.9 to 6.7]	−13.9 [−50.5 to 5.3]	−16.1 [−71.5 to 51.5]	−13.3 [−49.8 to 7.2]	−10.6 [−67 to 58.1]	−15.3 [−49.2 to 6.7]	2.4 [−47.6 to 78.6]

∗ CPUs, chest pain units; STEMI, ST elevation myocardial infarction.

**Fig. 2:**
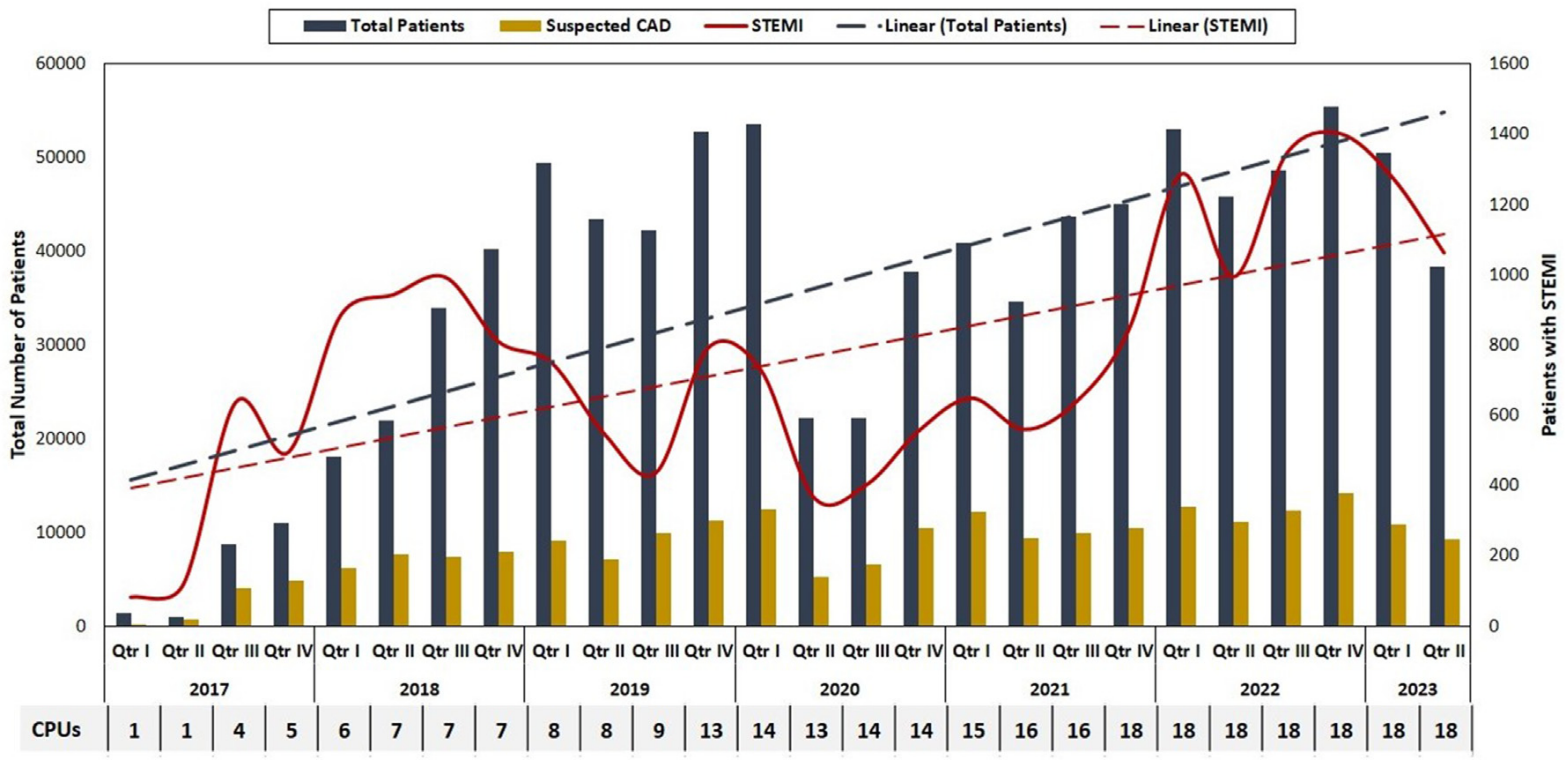
Trends in total number of patients seen at CPU, number of ST elevation myocardial infarction (STEMI) patients and patients with suspected coronary artery disease (CAD) between 2017 and 2023.

During the initial quarter of 2017, the CPUs attended to 1316 patients, including 82 with ST-segment elevation myocardial infarction (STEMI), 419 with cardiac concerns, and 897 with non-cardiac issues. In contrast, by 2023, the CPUs collectively managed 50,568 patients in the first quarter alone. This included 1401 STEMI patients, 20,078 cardiac patients, and 35,348 patients with non-cardiac conditions, marking a remarkable 17-fold increase in STEMI cases and a substantial 30-fold increase in the overall patient volume treated per quarter at CPUs (Fig. 2 and Supplemental Table S2).

### Patient characteristics and triage

Of the 0.915 million patients assessed at CPUs, 692,444 (75.6%) were categorized as non-cardiac and subsequently discharged from CPUs. A total of 223,120 (24.4%) patients were directed for additional care. The sex distribution showed 564,129 (61.6%) were males, and 351,435 (38.4%) were females, with 352,872 (38.5%) patients falling below the age of 40 (Table 1).

**Fig. 3:**
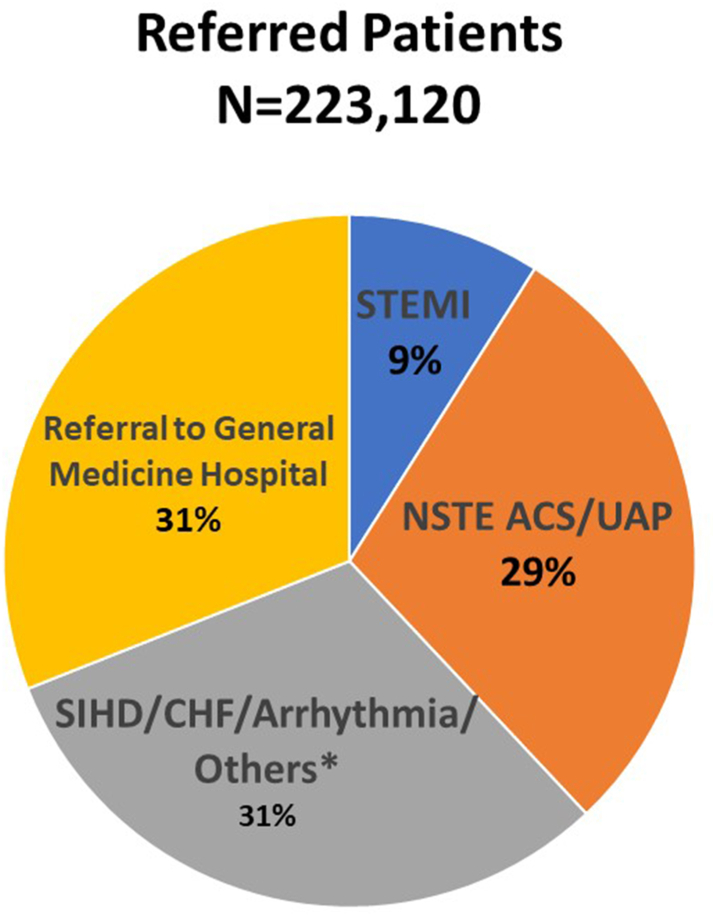
Pie Chart demonstrating distribution of referred patients for further evaluation and managements by the CPUs. ACS, acute coronary syndrome; CHF, congestive heart failure; NSTE, non ST elevation; SIHD, stable ischemic heart disease; STEMI, ST elevation myocardial infarction; UAP, unstable angina pectoris.

Among the referred patients, 9% were diagnosed with STEMI, 29% with Non-ST-segment elevation acute coronary syndrome (NSTE ACS)/unstable angina, and 31% presented with various other cardiac symptoms and conditions, including stable ischemic heart disease/angina, heart failure, arrhythmias, hypertensive urgency. Moreover, 31% of patients were directed to multidisciplinary tertiary care hospitals for suspected medical non-cardiac issues, undergoing further evaluation on an outpatient basis (Table 1; Fig. 3).

### Primary PCI of CPU referred STEMI patients

A total of 19,580 patients diagnosed with STEMI and referred from CPUs received primary PCI at the main center. Among these, 81% were male and 19% were female. The median age within this cohort was 56 years (Interquartile Range [IQR]: 50–65). Notably, 26% of STEMI patients were below 50 years old, and 9% were below 40 years old. Seven percent of patients had a prior history of MI, 18% were active smokers, 30% had diabetes, and 52% had hypertension. The mean body mass index (BMI) was 27.3 ± 4.2 kg/m^2^. Cardiogenic shock was observed in 1108 STEMI patients, constituting 5.66% of the cohort. The overall in-hospital mortality rate for patients undergoing primary PCI was 5.58%, with a range between 5.1% and 6.9% through the study period. Predictors of mortality included advanced age, female sex, prolonged total ischemic time, the presence of cardiogenic shock, increased creatinine and anterior wall infarct (Supplemental Table S3).

### Temporal presentation of STEMI at CPUs

The highest volume of STEMI cases at CPUs was observed between 6 pm and midnight (35%). Subsequent distributions were 24% from 12 pm to 6 pm, 23% from 6 am to 12 pm, and 18% from midnight until 6 am.

### Total ischemic time and FMC to device time

The median first medical contact (FMC) to device time was 100 min (IQR 80–135), revealing substantial variation contingent upon location. Specifically, eight CPUs were situated within a 30–60-min driving radius, whereas 10 were positioned beyond 60 min (Fig. 1). Total ischemic time likewise displayed a skewed distribution, with a median of 232 min (IQR: 172–315; 5th −95th %le: 50–920). Comparatively median door to balloon time was 84 min (IQR 60–125) for patients directly presenting to the NICVD ED.

### ACS diagnosis variation across CPUs

Among the patient cohort assessed at CPUs, approximately 2.13% received a diagnosis of STEMI while 6.9% were diagnosed with NSTE-ACS (Table 1). A notable variation was observed among the different CPU centers, with diagnostic rates for STEMI ranging from 0.4% to 3.2% (Fig. 1). Notably, CPU-5, situated at Nagan Chawrangi in the most densely populated zone of the city, exhibited the highest percentage of STEMI diagnoses at 3.2% (Fig. 1).

### Impact of CPUs on Cath lab volume

**Fig. 4:**
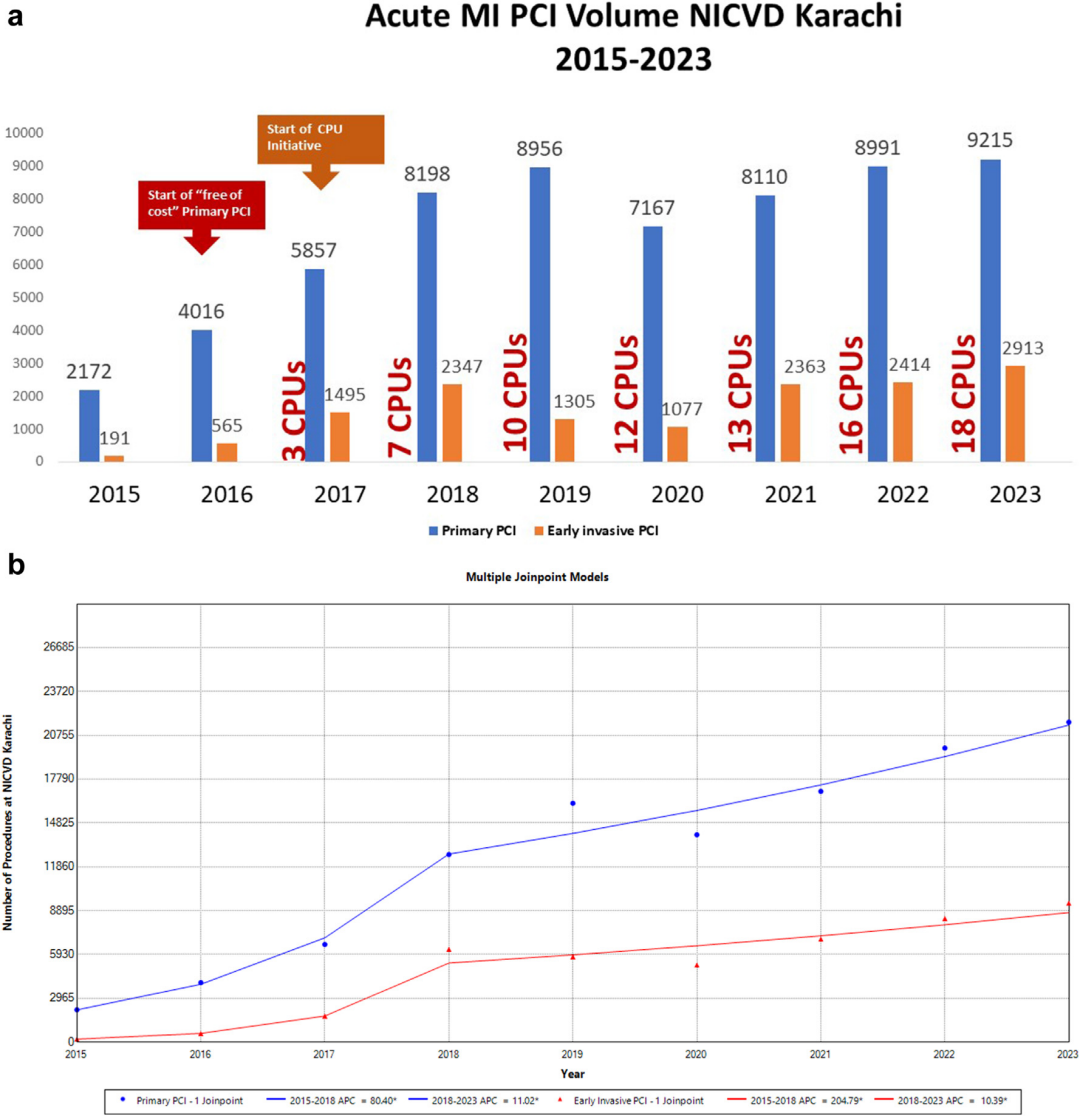
a. Trends in Primary PCI and early invasive PCI at NICVD Karachi between 2016 and 2023 and growth of CPU program (18 chest pain units). b: Average annual percentage change (AAPC) in primary PCI and early invasive PCI volume in Karachi over the study period.

The inception of CPUs concomitantly marked a notable increase in the caseload of primary PCI (for STEMI) and early invasive PCI procedures (for NSTE ACS) performed at NICVD. Demonstrating an annual growth rate ranging from 16% to 20%, the cumulative number of primary PCIs reached >9000 by 2023 (Fig. 4a). A commensurate escalation in the volume of early invasive PCI procedures was observed, mirroring the progressive growth and expansion of the CPU system throughout the city.

As presented in Fig. 4b, the AAPC in the volume of primary PCI procedures performed at NICVD. Karachi between 2015 and 2023 was 33.2 [95% CI: 30.3–35.9] with two distinct segments; 2015–2018 with AAPC of 80.4 [95% CI: 68.3–92] and 2018–2023 with AAPC of 11 [95% CI: 6.8–15.1]. Similarly, the AAPC in the volume of early invasive PCI procedures performed at NICVD Karachi between 2015 and 2023 was 61.6 [95% CI: 56.2–67.4] with two distinct segments; 2015–2018 with AAPC of 204.8 [95% CI: 176.7–240.4] and 2018–2023 with AAPC of 10.4 [95% CI: 3.7–16.6].

### Expansion of CPU and primary PCI services province-wide

**Fig. 5:**
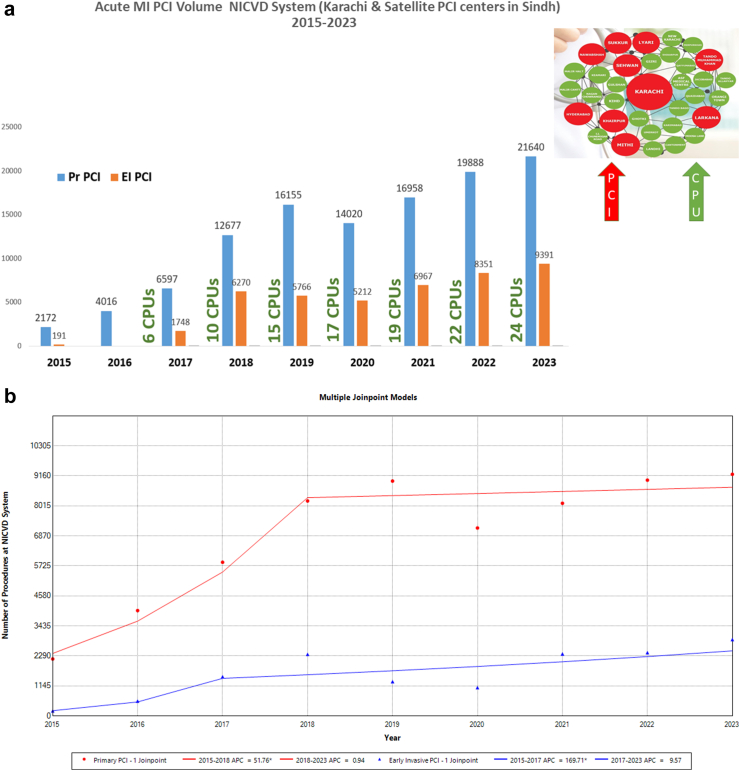
a. Trends in Primary PCI and early invasive PCI at NICVD Karachi & Satellite PCI centers^9^ in Sindh province between 2016 and 2023 and growth of CPU program (24 chest pain units). b: Average annual percentage change (AAPC) in primary PCI and early invasive PCI volume in NICVD health system across Sindh province over the study period.

This comprehensive effort has resulted in the strategic placement of 24 CPUs across the province (including the eighteen chest pain units and main cardiac center in Karachi), collectively attending to over 1.3 million patients to date. Notably, the total number of primary PCIs has reached 21,640, with an additional 9391 early invasive PCIs performed across the entire health system in 2023 (Fig. 5a). The geographical distribution of CPUs and PCI centers ensures that every region in Sindh is within a 120-min reach of a primary PCI facility. Fig. 5b visually portrays the comprehensive success of the program, depicting the total volume of PCIs conducted across the province's health system. As presented in Fig. 5b, the AAPC in the volume of primary PCI procedures performed at NICVD system between 2015 and 2023 was 17.6 [95% CI: 13.7–21.8] with two distinct segments; with marked increase from 2015 to 2018 with AAPC of 51.8 [95% CI: 34.9–78.5] followed by stability from 2018 to 2023 with AAPC of 0.9 [95% CI: −6.0 to 7.2]. Similarly, the AAPC in the volume of early invasive PCI procedures performed at NICVD system between 2015 and 2023 was 37.2 [95% CI: 24.3–50] with two distinct segments; 2015 to 2017 with AAPC of 169.7 [95% CI: 61.4–301.4] and 2017 to 2023 with insignificant AAPC of 9.6 [95% CI: −9.5 to 20.4].

## Discussion

NICVD's innovative implementation of stand-alone Chest Pain Units represents a paradigmatic shift in acute MI care within a densely populated setting in LMICs. These CPUs strategically placed in critical locations within a densely populated metropolis such as Karachi facilitated timely access for urgent evaluation, catering to a substantial cohort of 0.92 million patients over a 6-year time period. This approach proved highly effective in screening and triaging patients, resulting in an unprecedented surge in STEMI cases within the NICVD system. Furthermore, the identification of approximately 5% of patients with NSTE ACS, including unstable angina, underscores the CPUs' efficacy in recognizing diverse cardiac pathologies beyond the typical STEMI presentations. The observed median FMC to device time of 100 min, while acknowledging the substantial patient volume, aligns with acceptable standards. Inpatient mortality rates following Primary PCI ranging between 5 and 7% mirror those seen in developed countries, highlighting the clinical significance and success of the initiative in achieving comparable outcomes. Rapid diagnosis, timely intervention, and streamlined referral pathways collectively contributed to enhanced care quality and positive health outcomes.

Perhaps even more impactful is the potential of this model to serve as a blueprint for densely populated LMICs and healthcare systems globally. The units' effectiveness, portability, and strategic placement in high-density areas demonstrate an adaptable approach that can be tailored to unique contexts. The success of this initiative underscores the power of innovation in addressing complex healthcare challenges.

### Uniqueness of the chest pain unit model

A distinctive feature of the Karachi initiative lies in the strategic placement of stand-alone chest pain units. While rapid access chest pain centers, as established in the Western world such as the UK or Australia, play a vital role in expedited AMI care, they primarily operate within hospital premises to streamline emergency room flow.^9^, ^10^, ^11^ However, the current practice of admitting a large number of chest pain patients to the hospital leads to over triage and increased healthcare costs. Hence, the development of innovative chest pain units focusing on efficient triage and prompt treatment of acute cardiac complaints becomes imperative to enhance patient outcomes and streamline resource utilization. In contrast, the Karachi model extends beyond the hospital confines, immersing chest pain units within densely populated localities. These units, strategically situated under bridges or highways, in financial districts, market places and very densely populated slums “katchi abadis” where road access is difficult, offer a unique pathway for individuals experiencing acute chest pain to swiftly access timely diagnosis and triage. This pioneering approach breaks away from convention and does not currently exist anywhere else globally.

In the United States, chest pain accounts for over 7 million ED) visits annually, leading to significant overcrowding, inefficient utilization of healthcare resources, and delays in diagnosis, with associated costs exceeding $10 billion.^12^^,^^13^ The primary challenge lies in swiftly identifying patients with ACS or other life-threatening conditions among the multitude presenting with benign or non-cardiac symptoms.^12^, ^13^, ^14^

**Fig. 6:**
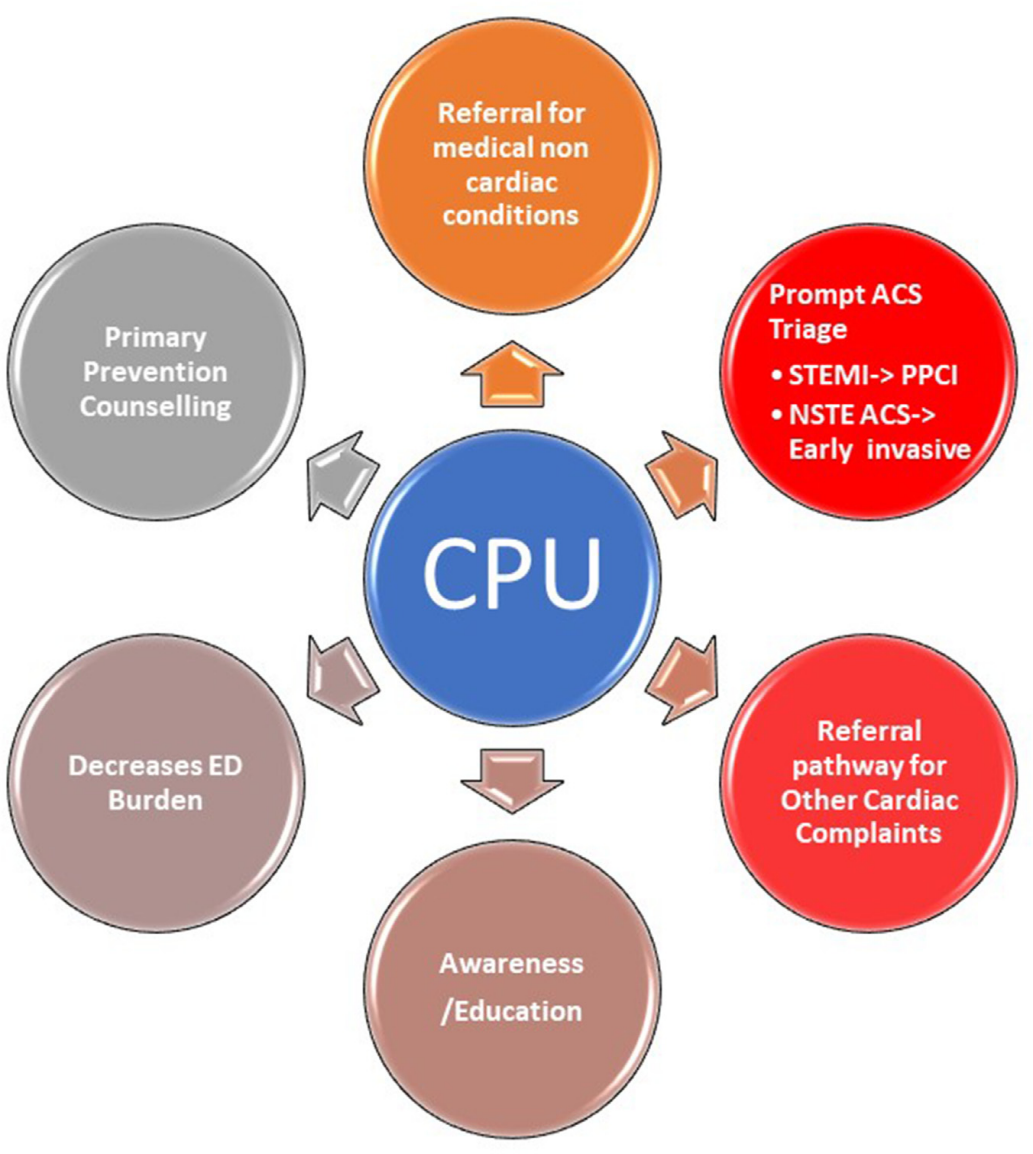
Comprehensive roles of the CPU program.

Against this backdrop, the NICVD's Chest Pain Units have implemented an effective triage system, categorizing 75.6% of cases as non-urgent and facilitating their discharge. This system has serviced a broad demographic, comprising 61% males and 39% females, with 38% of the patients under 40 years of age. Among those referred for further care, 9% were diagnosed with STEMI, 29% with NSTE ACS, and 31% presented with a spectrum of other cardiac conditions. Furthermore, 31% of individuals were evaluated for non-cardiac issues, underscoring the CPUs' comprehensive assessment capabilities. This strategic approach has likely mitigated the impact on the already overburdened and overcrowded emergency rooms across the city, demonstrating a pivotal shift in managing emergency cardiac care (Fig. 6).

### Prompt ACS diagnosis, triage and timely reperfusion

The assessment of the Karachi CPU model's effectiveness is ideally framed through a comparison of primary reperfusion rates before and after its implementation. Unfortunately, due to the absence of historical data on STEMI primary reperfusion and outcomes in the city, a comprehensive evaluation is not possible. Nevertheless, the observed reperfusion rate, with a FMC to device time of 100 min, juxtaposed against a DTB of 80 min for direct ED presentations, coupled with over 95% of patients undergoing primary PCI within 12 h of symptom onset and a mortality range of 5–7%, closely mirrors rates reported in international datasets.^15^

The successful referral and subsequent Primary PCI for STEMI patients highlight the CPUs' role in expediting critical cardiac interventions. Predictors of mortality, such as advanced age and prolonged total ischemic time, underscore the significance of swift diagnosis and intervention for enhanced patient outcomes. While comprehensive analysis faced challenges, the evaluation of total ischemic time and first medical contact to device time underscores the notable impact of CPU locations on these crucial metrics.

The increase in primary PCI and early invasive PCI procedures at NICVD correlates with CPU growth, strongly suggesting a positive trend in expediting reperfusion strategies. The temporal concentration of STEMI cases between 6 pm and midnight emphasizes the necessity for resource allocation and staffing considerations during peak hours.

### Global implications

The global impact of the Karachi chest pain unit model reverberates across healthcare landscapes. Unlike conventional rapid access centers that optimize existing structures, the stand-alone units represent a paradigm shift. Their immediate ECG access and streamlined referrals address the unique challenges faced by densely populated LMICs. The Karachi initiative, as a potential blueprint, offers transformative possibilities for AMI care worldwide, ensuring equitable access and improved outcomes. Rooted in strategic placement, expedited triage, and timely management, it stands poised to meet the distinctive healthcare challenges of South and East Asian nations, heralding a transformative era in AMI care. Serving as a model thriving within resource constraints, the chest pain unit initiative encourages global healthcare systems to rethink their approaches. The collaborative effort involving government bodies, healthcare professionals, and local communities exemplifies a holistic approach transcending boundaries and leveraging collective strengths. Ongoing electronic medical records integration underscores the initiative's dynamism, aiming to expedite transfers through real-time patient information exchange between units and ambulance services.

This report encounters notable limitations, primarily stemming from the absence of comprehensive pre-initiative data, impairing a robust pre-post analysis of the CPU model's efficacy. The NICVD caters to over 90% of the STEMI incidents in Karachi, yet there are ten additional hospitals providing fee-based PPCI services, collectively managing around 800 cases per year. Unfortunately, specific patient details from these institutions remain elusive, with an estimated 20% of STEMI cases in these private settings purportedly originating from the CPU network. Significant variation in ACS prevalence across CPU locations (0.7–3.2%) remains unexplored for underlying factors. While definitive conclusions cannot be drawn without further investigation, several potential factors may contribute to this variation. These include variances in socioeconomic status, a potentially higher burden of underlying risk factors, such as hypertension and diabetes, and variations in healthcare-seeking behavior. Additionally, factors such as overcrowding and differing levels of access to healthcare resources across different CPU locations may also influence the rates of ACS diagnosis. Further research is necessary to explore these hypotheses and determine the underlying factors contributing to the observed variation in ACS diagnosis rates among CPUs. The inclusive nature of time metrics analysis, encompassing patients in cardiogenic shock, lacks granularity on relative delays and contributing factors. Notably, the study does not address the crucial aspect of cost-effectiveness, which is imperative for understanding the economic implications of the CPU model compared to potential healthcare savings and public health improvements. While we recognize the importance of long-term clinical outcomes such as mortality beyond hospital discharge, recurrent myocardial infarction, and quality of life post-treatment, these data have not been systematically captured in the current study. These limitations underscore the need for future research to comprehensively address these aspects, considering the potential impact of the CPU model on both clinical outcomes and healthcare economics.

## Contributors

NQ, AH: Conceptualization, data curation, supervision, formal analysis; writing original draft review & editing. JS, TS, ZH, AZS, KK, JAS, SK, SB: Data curation, validation, writing review & editing. MK: Data curation, statistics, editing. AH, NQ, ZH and AZS have directly accessed and verified the underlying data. All authors agreed with the final version of the manuscript.

## Data sharing statement

De-identified data collected for this study are available upon a reasonable request over email to the corresponding author.

## Declaration of interests

None.

## Figures and Tables

**Supplemental Figure S1a:**
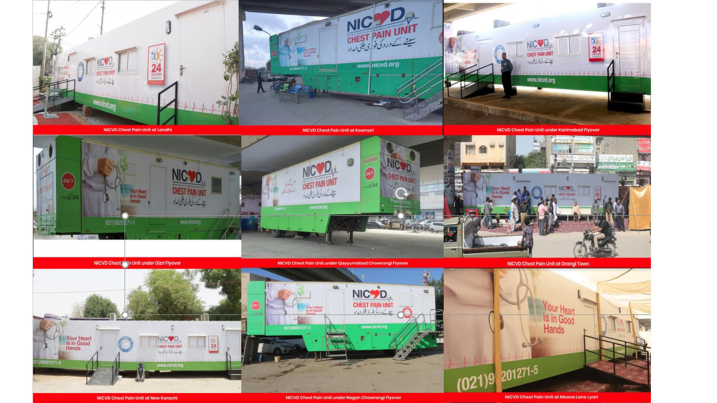


**Supplemental Figure S1b:**
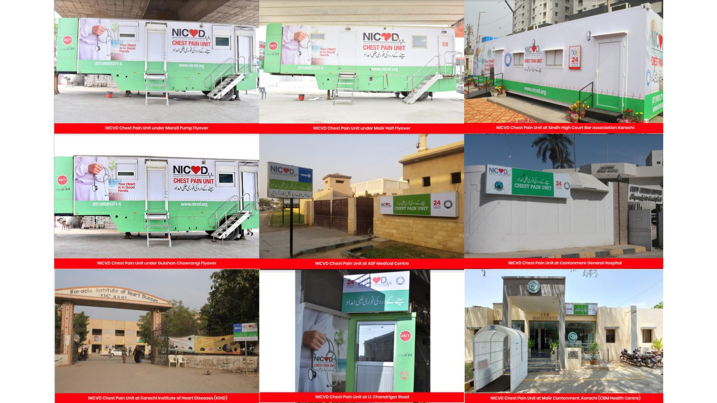


## Appendix A Supplementary data

Supplemental Table S3

CPU video
