# Supplementary material for: Advancing acute MI care in densely populated low- and middle-income countries (LMICs): innovative stand-alone chest pain units for expedited triage and timely management
Source: Lancet Reg Health Southeast Asia. 2024 Sep 30;30:100488. doi: 10.1016/j.lansea.2024.100488 (PMC11474207; doi:10.1016/j.lansea.2024.100488)
Supplement: Supplemental Table S1 [file mmc1.docx]

**Supplementary Table 1a: NICVD Satellite Primary PCI Centers**

|  | **Center Name** | **Establishment date** |
| --- | --- | --- |
| 1. | NICVD Larkana | 13^th^ May, 2017 |
| 2. | NICVD Tando Muhammad Khan | 19^th^ October, 2017 |
| 3. | NICVD Hyderabad | 25^th^ November, 2017 |
| 4. | NICVD Sehwan | 29^th^ December, 2017 |
| 5. | NICVD Sukkur | 24^th^ February, 2017 |
| 6. | NICVD Nawabshah | 12^th^ April, 2018 |
| 7. | NICVD Mithi | 6^th^ May, 2018 |
| 8. | NICVD Khairpur | 15^th^ May, 2018 |
| 9. | NICVD Lyari | 3^rd^ October, 2019 |

**Supplementary Table 1bL NICVD Health System CPUs**

|  | **Center Name** | **Establishment date** |
| --- | --- | --- |
| 1. | NICVD CPU under Gulshan Chowrangi Flyover, Karachi | 8^th^ May, 2017 |
| 2. | NICVD CPU under Malir Halt Flyover, Karachi | 14^th^ July, 2017 |
| 3. | NICVD CPU under Qayyumabad Chowrangi Flyover | 18^th^ July , 2017 |
| 4. | NICVD CPU under Nagan Chowrangi Flyover, Karachi '. | 22^nd^ November, 2017 |
| 5. | NICVD CPU near I.I. Chandrigar Road, Karachi | 3^rd^ January, 2018 |
| 6. | NICVD CPU at Landhi 3 1/2, Karachi, Karachi | 15^th^ January, 2019 |
| 7. | NICVD CPU under Karimabad Flyover, Karachi | 19^th^ April, 2019 |
| 8. | NICVD CPU at Ghotki | 24^th^ June, 2019 |
| 9. | NICVD CPU at Tando Sago (Badin) | 27^th^ July, 2019 |
| 10. | NICVD CPU at Orangi Town, Karachi | 17^th^ September, 2019 |
| 11. | NICVD CPU under Gizri Flyover , Karachi | 3^rd^ October, 2019 |
| 12. | NICVD CPU at New Karachi | 15^th^ October, 2019 |
| 13. | 'NICVD CPU at Moosa Lane, Lyari, Karachi | 14^th^ November, 2019 |
| 14. | NICVD CPU at Quaidabad, Karachi | 15^th^ December, 2019 |
| 15. | NICVD CPU Jacobabad | 23^rd^ January, 2020 |
| 16. | NICVD CPU at Cantonment General Hospital, Karachi | 27^th^ February, 2020 |
| 17. | NICVD CPU Umerkot, at Faqir Abadullah Dialysis Center | 12^th^ July, 2020 |
| 18. | NICVD CPU Keamari, Karachi | 31^st^ July, 2020 |
| 19. | NICVD CPU ASF, Medical Centre, Karachi | 1^st^ February, 2021 |
| 20. | CPU Tando Allahyar (DI-IQ Hospital) | 1^st^ April, 2021 |
| 21. | NICVD CPU Malir Cantonment, Karachi | 19^th^ April, 2021 |
| 22. | NICVD CPU K1HD, Karachi | 24^th^ December, 2021 |
| 23. | NICVD CPU Mirpurkhas | 5^th^ February, 2022 |
| 24. | NICVD CPU Shikarpur | 16^th^ April, 2022 |
|  |  |  |
