# Supplementary material for: Advancing acute MI care in densely populated low- and middle-income countries (LMICs): innovative stand-alone chest pain units for expedited triage and timely management
Source: Lancet Reg Health Southeast Asia. 2024 Sep 30;30:100488. doi: 10.1016/j.lansea.2024.100488 (PMC11474207; doi:10.1016/j.lansea.2024.100488)
Supplement: Supplemental Table S2 [file mmc2.docx]

**Supplemental Table 2: Patient Volume and Characteristics at CPUs**

|  | **Total Patients** | **Gender** | | **Age** | | | **Type** | | **Disposition** | | **STEMI** |
| --- | --- | --- | --- | --- | --- | --- | --- | --- | --- | --- | --- |
|  |  | **Male** | **Female** | **≤ 40 years** | **41 to 65 years** | **>65 years** | **Cardiac** | **Non-cardiac** | **Referred** | **Discharge** |  |
| **Total** | **915,564** | **564,129** | **351,435** | **352,872** | **485,102** | **77,590** | **306,794** | **608,770** | **223,120** | **692,444** | **19,580** |
| Qtr I, 2017 | 1,316 | 1,028 | 288 | 512 | 703 | 101 | 419 | 897 | 187 | 1,129 | 82 |
| Qtr II, 2017 | 980 | 781 | 199 | 370 | 523 | 87 | 253 | 727 | 738 | 239 | 117 |
| Qtr III, 2017 | 8,641 | 6,385 | 2,256 | 3,210 | 4,678 | 753 | 3,644 | 4,997 | 4,043 | 4,601 | 636 |
| Qtr IV, 2017 | 10,920 | 7,880 | 3,040 | 4,067 | 5,919 | 934 | 5,499 | 5,421 | 4,849 | 6,071 | 494 |
| Qtr I, 2018 | 17,989 | 12,648 | 5,341 | 7,155 | 9,296 | 1,538 | 8,306 | 9,683 | 6,202 | 11,787 | 884 |
| Qtr II, 2018 | 21,971 | 14,617 | 7,354 | 8,127 | 11,936 | 1,908 | 9,906 | 12,065 | 7,640 | 14,331 | 942 |
| Qtr III, 2018 | 33,945 | 20,031 | 13,914 | 13,417 | 17,873 | 2,655 | 10,198 | 23,747 | 7,410 | 26,535 | 991 |
| Qtr IV, 2018 | 40,188 | 24,115 | 16,073 | 16,535 | 20,635 | 3,018 | 11,885 | 28,303 | 7,881 | 32,307 | 809 |
| Qtr I, 2019 | 49,479 | 30,322 | 19,157 | 19,351 | 25,821 | 4,307 | 15,543 | 33,936 | 9,055 | 40,424 | 750 |
| Qtr II, 2019 | 43,420 | 26,805 | 16,615 | 16,910 | 22,493 | 4,017 | 13,189 | 30,231 | 7,102 | 36,318 | 549 |
| Qtr III, 2019 | 42,198 | 25,010 | 17,188 | 16,419 | 22,540 | 3,239 | 11,883 | 30,315 | 9,933 | 32,265 | 436 |
| Qtr IV, 2019 | 52,717 | 32,080 | 20,637 | 19,885 | 28,116 | 4,716 | 14,642 | 38,075 | 11,206 | 41,511 | 792 |
| Qtr I, 2020 | 53,585 | 33,621 | 19,964 | 19,903 | 29,011 | 4,671 | 16,750 | 36,835 | 12,509 | 41,076 | 727 |
| Qtr II, 2020 | 22,230 | 13,982 | 8,248 | 8,278 | 12,050 | 1,902 | 8,264 | 13,966 | 5,235 | 16,995 | 366 |
| Qtr III, 2020 | 22,133 | 14,126 | 8,007 | 8,804 | 11,437 | 1,892 | 9,243 | 12,890 | 6,508 | 15,625 | 401 |
| Qtr IV, 2020 | 37,795 | 24,220 | 13,575 | 13,980 | 20,533 | 3,282 | 15,225 | 22,570 | 10,448 | 27,347 | 555 |
| Qtr I, 2021 | 40,916 | 26,275 | 14,641 | 16,173 | 21,543 | 3,200 | 15,315 | 25,601 | 12,129 | 28,787 | 648 |
| Qtr II, 2021 | 34,577 | 22,295 | 12,282 | 14,226 | 17,754 | 2,597 | 12,439 | 22,138 | 9,372 | 25,205 | 559 |
| Qtr III, 2021 | 43,658 | 27,246 | 16,412 | 17,074 | 22,783 | 3,801 | 14,847 | 28,811 | 9,965 | 33,693 | 638 |
| Qtr IV, 2021 | 45,003 | 27,133 | 17,870 | 17,527 | 23,313 | 4,163 | 16,152 | 28,851 | 10,381 | 34,622 | 843 |
| Qtr I, 2022 | 53,005 | 32,294 | 20,711 | 20,624 | 28,313 | 4,068 | 18,300 | 34,705 | 12,687 | 40,318 | 1,285 |
| Qtr II, 2022 | 45,882 | 27,319 | 18,563 | 17,307 | 24,471 | 4,104 | 14,922 | 30,960 | 11,129 | 34,753 | 993 |
| Qtr III, 2022 | 48,644 | 28,023 | 20,621 | 18,068 | 26,336 | 4,240 | 17,076 | 31,568 | 12,284 | 36,360 | 1,343 |
| Qtr IV, 2022 | 55,426 | 32,323 | 23,103 | 20,640 | 30,044 | 4,742 | 20,078 | 35,348 | 14,144 | 41,282 | 1,401 |
| Qtr I, 2023 | 50,568 | 31,049 | 19,519 | 20,114 | 26,131 | 4,323 | 12,981 | 37,587 | 10,818 | 39,750 | 1,278 |
| Qtr II, 2023 | 38,378 | 22,521 | 15,857 | 14,196 | 20,850 | 3,332 | 9,835 | 28,543 | 9,265 | 29,113 | 1,061 |
