# Supplementary material for: Advancing acute MI care in densely populated low- and middle-income countries (LMICs): innovative stand-alone chest pain units for expedited triage and timely management
Source: Lancet Reg Health Southeast Asia. 2024 Sep 30;30:100488. doi: 10.1016/j.lansea.2024.100488 (PMC11474207; doi:10.1016/j.lansea.2024.100488)
Supplement: Supplemental Table S3 [file mmc3.docx]

**Table: Univariable and multivariable binary logistic regression analysis for in-hospital mortality**

|  | **Univariable** | | ***Multivariable - Initial Solution** | | ***Multivariable - Final Solution** | |
| --- | --- | --- | --- | --- | --- | --- |
|  | **OR [95% CI]** | **P-value** | **OR [95% CI]** | **P-value** | **OR [95% CI]** | **P-value** |
| Female | 1.91 [1.55 - 2.36] | <0.001 | 1.58 [1.01 - 2.49] | 0.046 | 1.64 [1.1 - 2.45] | 0.015 |
| Age (year) | 1.05 [1.04 - 1.06] | <0.001 | 1.04 [1.02 - 1.06] | <0.001 | 1.04 [1.02 - 1.06] | <0.001 |
| Body mass index (kg/m2) | 1 [0.97 - 1.02] | 0.803 | 0.99 [0.94 - 1.03] | 0.591 | - | - |
| Symptom onset to Arrival Time (hrs) | 1.046 [1.023 - 1.071] | <0.001 | 1.055 [1.012 - 1.099] | 0.011 | 1.054 [1.012 - 1.097] | 0.011 |
| Door to Balloon Time (hrs) | 1.04 [1.01 - 1.07] | 0.017 | 1 [0.94 - 1.08] | 0.889 | - | - |
| Hypertension | 1.75 [1.43 - 2.14] | <0.001 | 1.75 [1.16 - 2.66] | 0.008 | 1.81 [1.21 - 2.72] | 0.004 |
| Diabetes | 2.12 [1.74 - 2.57] | <0.001 | 1.17 [0.79 - 1.73] | 0.425 | - | - |
| History of MI | 1.69 [1.24 - 2.32] | <0.001 | 1.3 [0.52 - 3.25] | 0.577 | 0.48 [0.21 - 1.08] | 0.074 |
| History of CVD | 3.17 [1.77 - 5.65] | <0.001 | 0.07 [0 - 2.43] | 0.141 | - | - |
| History of HF | 7.8 [5.76 - 10.56] | <0.001 | 0.53 [0.23 - 1.18] | 0.119 | - | - |
| Prior PCI | 1.44 [1.01 - 2.04] | 0.043 | 0.68 [0.23 - 1.98] | 0.483 | - | - |
| Every day tobacco user | 0.48 [0.34 - 0.68] | <0.001 | 1.1 [0.6 - 2.02] | 0.766 | - | - |
| Cardiac Arrest Out of Hospital | 8.1 [1.48 - 44.37] | 0.016 | 18.42 [1.27 - 268.11] | 0.033 | 18.8 [1.32 - 267.91] | 0.03 |
| Cardiac Arrest In Hospital | 588 [330.73 - 1045.37] | <0.001 | 503.24 [232.98 - 1086.97] | <0.001 | 498.75 [232.98 - 1067.71] | <0.001 |
| Ventricular fibrillation (VF) | 9.75 [2.32 - 40.91] | 0.002 | 1.34 [0.02 - 94.61] | 0.893 | 0.06 [0.01 - 0.71] | 0.025 |
| Sustained VT | 13.6 [4.13 - 44.73] | <0.001 | 0.09 [0.01 - 1.49] | 0.093 | - | - |
| 3rd Degree AV Heart Block | 7.54 [2.85 - 19.93] | <0.001 | 0.15 [0.01 - 1.79] | 0.135 | - | - |
| Femoral access | 8.68 [7.07 - 10.66] | <0.001 | 3.58 [2.4 - 5.36] | <0.001 | 3.64 [2.46 - 5.38] | <0.001 |
| Venous Access | 15.49 [9.58 - 25.02] | <0.001 | 2.02 [0.73 - 5.63] | 0.178 | - | - |
| Systolic BP (mmHg) | 0.96 [0.96 - 0.97] | <0.001 | 0.99 [0.98 - 1] | 0.003 | 0.99 [0.98 - 0.99] | 0.001 |
| Fluoroscopy Time (min) | 1.03 [1.02 - 1.04] | <0.001 | 1.02 [1 - 1.03] | 0.017 | 1.02 [1 - 1.03] | 0.019 |
| Contrast Volume (mL) | 1.003 [1.001 - 1.006] | 0.002 | 0.999 [0.995 - 1.004] | 0.797 | - | - |
| Pre-proc creatinine (mg/dL) | 2.47 [2.12 - 2.88] | <0.001 | 1.86 [1.53 - 2.26] | <0.001 | 1.87 [1.54 - 2.26] | <0.001 |
| HGB (g/dL) | 0.86 [0.82 - 0.91] | <0.001 | 0.99 [0.9 - 1.1] | 0.922 | - | - |
| CV Instability | 8.39 [6.73 - 10.45] | <0.001 | 1.96 [1.18 - 3.26] | 0.009 | 2.01 [1.22 - 3.32] | 0.006 |
| Ventricular Support | 26.36 [20.93 - 33.2] | <0.001 | 5.55 [1.16 - 26.6] | 0.032 | 7.35 [4.43 - 12.2] | <0.001 |
| Pressor Support | 22.15 [17.49 - 28.07] | <0.001 | 1.33 [0.3 - 5.9] | 0.706 | - | - |
| Mech Vent Support | 62.95 [39.68 - 99.86] | <0.001 | 3.91 [1.26 - 12.17] | 0.019 | 3.55 [1.5 - 8.42] | 0.004 |
| MV Support Device (IABP) | 62.95 [39.68 - 99.86] | <0.001 | - | - | - | - |
| **Vessels involved** |  |  |  |  |  |  |
| LAD | 2.76 [1.99 - 3.83] | <0.001 | 2.13 [1.07 - 4.26] | 0.031 | 1.98 [1.13 - 3.49] | 0.017 |
| RCA | 1.84 [1.51 - 2.25] | <0.001 | 1.3 [0.77 - 2.19] | 0.327 | - | - |
| LCX | 2 [1.64 - 2.45] | <0.001 | 1.33 [0.75 - 2.35] | 0.331 | 1.42 [0.96 - 2.09] | 0.077 |
| LM | 5.22 [3.82 - 7.13] | <0.001 | 1.96 [0.98 - 3.93] | 0.057 | 1.98 [1 - 3.93] | 0.051 |
| Multi-vessel disease | 2.82 [2.26 - 3.53] | <0.001 | 1 [0.45 - 2.21] | 0.999 | - | - |
| **Pre-procedure TIMI flow** |  |  |  |  |  |  |
| 0 | 1.95 [1.3 - 2.94] | 0.001 | 1.63 [0.77 - 3.44] | 0.198 | 1.6 [0.76 - 3.37] | 0.216 |
| I | 1.54 [0.94 - 2.53] | 0.086 | 0.87 [0.33 - 2.31] | 0.779 | 0.84 [0.32 - 2.25] | 0.735 |
| II | 0.86 [0.52 - 1.4] | 0.535 | 0.76 [0.31 - 1.88] | 0.559 | 0.76 [0.31 - 1.86] | 0.548 |
| III | Reference | - | Reference | - | Reference | - |
| High/C | 1.69 [1.38 - 2.07] | <0.001 | 0.64 [0.38 - 1.08] | 0.094 | - | - |
| Lesion Length (mm) | 1.03 [1.02 - 1.04] | <0.001 | 1.04 [1.01 - 1.06] | 0.003 | 1.02 [1 - 1.04] | 0.012 |
| Severe Calcification | 1.58 [1.28 - 1.94] | <0.001 | 0.85 [0.54 - 1.31] | 0.456 | - | - |
| Bifurcation Lesion | 1.84 [1.46 - 2.31] | <0.001 | 1.6 [1.01 - 2.54] | 0.047 | 1.48 [0.95 - 2.32] | 0.085 |

**Backward conditional variable selection*

*CVD= Cardiovascular disease; IABP= intra aortic balloon pump; LAD= left anterior descending ; LM= left main; LCX= left circumflex; MI= myocardial infarction; OR= odds Ratio; RCA= right coronary artery*
